# Cytoplasmic Autoinhibition in HCN Channels is Regulated by the Transmembrane Region

**DOI:** 10.1007/s00232-020-00111-8

**Published:** 2020-03-07

**Authors:** Dana A. Page, Kaylee E. A. Magee, Jessica Li, Matthew Jung, Edgar C. Young

**Affiliations:** 1 Department of Molecular Biology and Biochemistry, Simon Fraser University, 8888 University Drive, Burnaby, BC V5A 1S6 Canada; 2 Department of Biology, Kwantlen Polytechnic University, 12666 72 Avenue, Surrey, BC V3W 2M8 Canada

**Keywords:** HCN channel, cAMP, Autoinhibition, Kinetics, Deactivation, Activation

## Abstract

Hyperpolarization-activated cation-nonselective (HCN) channels regulate electrical activity in the brain and heart in a cAMP-dependent manner. The voltage-gating of these channels is mediated by a transmembrane (TM) region but is additionally regulated by direct binding of cAMP to a cyclic nucleotide-binding (CNB) fold in the cytoplasmic C-terminal region. Cyclic AMP potentiation has been explained by an autoinhibition model which views the unliganded CNB fold as an inhibitory module whose influence is disrupted by cAMP binding. However, the HCN2 subtype uses two other CNB fold-mediated mechanisms called open-state trapping and Quick-Activation to respectively slow the deactivation kinetics and speed the activation kinetics, against predictions of an autoinhibition model. To test how these multiple mechanisms are influenced by the TM region, we replaced the TM region of HCN2 with that of HCN4. This HCN4 TM-replacement preserved cAMP potentiation but augmented the magnitude of autoinhibition by the unliganded CNB fold; it moreover disrupted open-state trapping and Quick-Activation so that autoinhibition became the dominant mechanism contributed by the C-terminal region to determine kinetics. Truncation within the CNB fold partially relieved this augmented autoinhibition. This argues against the C-terminal region acting like a portable module with consistent effects on TM regions of different subtypes. Our findings provide evidence that functional interactions between the HCN2 TM region and C-terminal region govern multiple CNB fold-mediated mechanisms, implying that the molecular mechanisms of autoinhibition, open-state trapping, and Quick-Activation include participation of TM region structures.

**Electronic supplementary material:**

The online version of this article (10.1007/s00232-020-00111-8) contains supplementary material, which is available to authorized users.

## Introduction

Hyperpolarization-activated cation-nonselective (HCN) channels produce the *I*_h_ or *I*_f_ "pacemaker" currents that regulate rhythmic firing in the brain and heart (reviewed in Wahl-Schott and Biel 2009). HCN channels are activated by membrane hyperpolarization, leading to an inward mixed Na^+^/K^+^ current which promotes action potential initiation. This hyperpolarization-activation is potentiated by direct binding of cytosolic cAMP such that the *V*_1/2_ value (the midpoint voltage of the conductance–voltage relation) is positively shifted, activation kinetics are speeded, and deactivation kinetics are slowed (DiFrancesco and Tortora 1991; Ludwig et al. 1998, 1999; Santoro et al. 1998). This cAMP-dependent activity is proposed to regulate thalamocortical oscillations associated with sleep states and epileptic seizures (Bal and McCormick 1996) as well as heartbeat pacing in the sinoatrial node (DiFrancesco 1986). The transmembrane (TM) region of HCN channels is homologous to the tetrameric Kv channel superfamily with six transmembrane helices (S1–S6), where S1 through S4 form a voltage-sensing domain with a positively charged mobile S4 voltage sensor, and S5 through S6 form a pore domain (Wahl-Schott and Biel 2009). Cyclic AMP potentiation is mediated by a large cytoplasmic C-terminal region which includes a C-linker with multiple helices, a cyclic nucleotide-binding (CNB) fold homologous to that found in protein kinase A, and an extreme C-terminal region (Zagotta et al. 2003). The C-linker and CNB fold act together as an independently folded domain undergoing cAMP-induced conformational changes (Zagotta et al. 2003; Zhou et al. 2004; Taraska et al. 2009; Akimoto et al. 2014; VanSchouwen et al. 2015; Goldschen-Ohm et al. 2016), but there is incomplete understanding of molecular events by which this "cAMP-sensing domain" would modify the energetics and kinetics of voltage-gating.

HCN channels have evolved multiple regulatory functions for the CNB fold affecting distinct voltage-gating parameters (Wicks et al. 2009, 2011; Magee et al. 2015), which have been uncovered in studies of HCN channels truncated to delete the CNB fold ("ΔCNB" derivatives). The cAMP-dependent positive *V*_1/2_ shift has been explained by an "autoinhibition" model analogous to that of protein kinase A: the presence of the unliganded CNB fold inhibits channel activity causing a negative *V*_1/2_ shift relative to the ΔCNB channel (that is, an "autoinhibitory *V*_1/2_ shift"), and cAMP binding then relieves this autoinhibition (Barbuti et al. 1999; Wainger et al. 2001). The autoinhibition model envisions that a ΔCNB channel has maximally favoured hyperpolarization-activation energetics, and applying a Leffler-type transition state model (Leffler 1953) would predict that CNB fold deletion should additionally enable maximally fast hyperpolarization-dependent activation kinetics and maximally slow depolarization-dependent deactivation. But these autoinhibition-based predictions are notably contradicted for kinetics of at least one HCN subtype, mouse HCN2 (Magee et al. 2015). For instance, cAMP-liganded HCN2 follows an "open-state trapping" model in its deactivation kinetics, with slower deactivation than the ΔCNB channel (Wicks et al. 2009, 2011; Magee et al. 2015). And even when unliganded and hence autoinhibited with a hyperpolarized *V*_1/2_, HCN2 follows a "Quick-Activation" model in its activation kinetics, with faster activation than the ΔCNB channel (Magee et al. 2015). Moreover, HCN channels have a multi-step gating pathway where voltage-dependent S4 movement occurs separately from voltage-independent pore opening (Craven and Zagotta 2004; Chen et al. 2007), with hysteresis such that the deactivation pathway is not the reverse of the activation pathway (Männikkö et al. 2005; Wicks et al. 2009; Kusch et al. 2010). Therefore, a full understanding of HCN channel gating requires elucidation of multiple, co-existing mechanisms that all depend on the CNB fold, and yet have different structural determinants and target rate-limiting reaction steps of distinct pathways.

Cross-linking and cryoEM studies have shown physical proximity of the C-linker and TM region (Decher et al. 2004; Prole and Yellen 2006; Kwan et al. 2012; Lee and MacKinnon 2017, 2019), but did not clarify how significant this proximity would be functionally for the multiple CNB fold-mediated regulatory mechanisms. This is especially true for autoinhibition which can be evaluated only through comparison with a ΔCNB channel, and for deactivation kinetics which have been less commonly studied in HCN channels. In this study, we report evidence for functional interaction between the TM region and C-terminal region in governing CNB fold-mediated mechanisms. We replaced the HCN2 TM region with that of HCN4 and found that the magnitude of the autoinhibitory *V*_1/2_ shift imposed by the unliganded CNB fold was significantly augmented. Further, the open-state trapping and Quick-Activation mechanisms characteristic of HCN2 were disrupted such that the augmented autoinhibition became the dominant mechanism contributed by the HCN2 C-terminal region to determine kinetics for both deactivation and activation. This establishes that interaction with the TM region supports the complex control of both thermodynamics and kinetics by the HCN2 C-terminal region.

## Results

### HCN4 TM-Replacement Preserves cAMP-Dependent ***V***_1/2_ Shift but Augments Autoinhibitory ***V***_1/2_ Shift

**Fig. 1:**
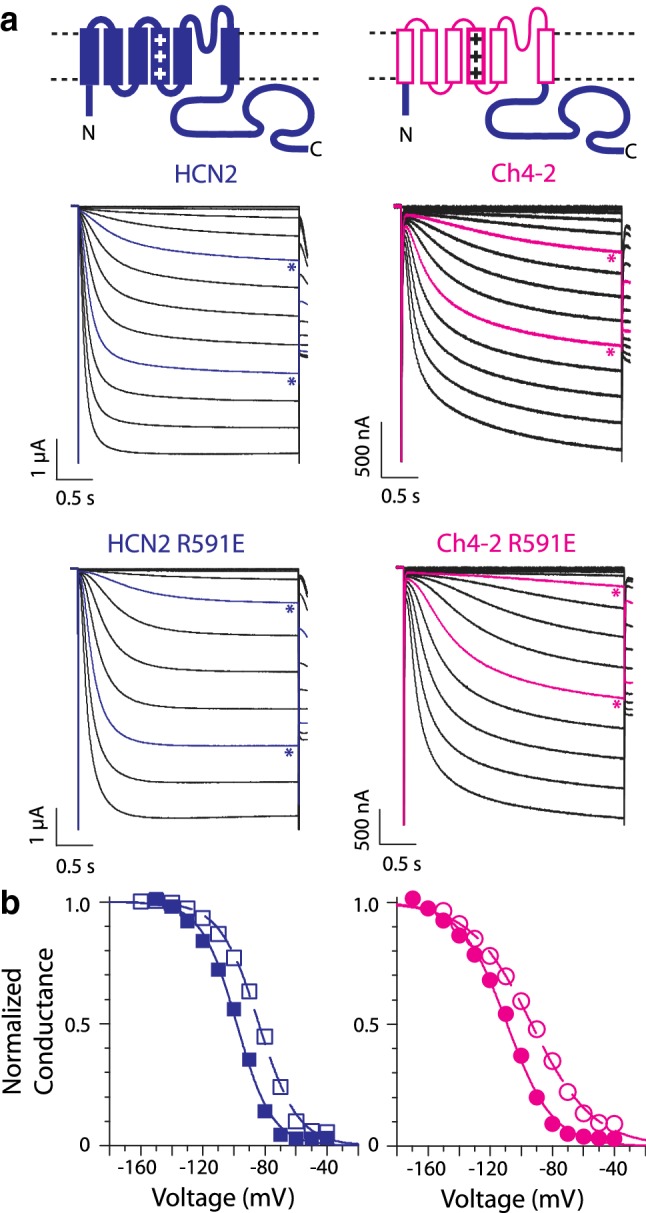
HCN4 TM-replacement in HCN2 inhibits activation but preserves cAMP-dependent *V*_1/2_ shift. **a** Schematics: composition of HCN2 and the chimeric Ch4-2 channel produced by HCN4 TM-replacement in HCN2. *Rectangles* represent transmembrane helices S1 through S6. HCN2 sequence is shown in *thick curves* and *solid rectangles*; HCN4 sequence is shown in *thin curves* and *open rectangles*. *Traces:* Representative inward currents of HCN2 and Ch4-2 (*upper*) and their corresponding R591E mutants lacking cAMP binding (*lower*). Recordings of HCN2 family channels are from Magee et al. (2015). Channel currents were elicited by step hyperpolarizations to various voltages followed by a tail epoch at − 120 mV for *V*_1/2_ determination. In each dataset, *stars* indicate traces for − 90 mV and − 130 mV. **b** Conductance–voltage relationships for HCN2 (*open squares*) compared to HCN2 R591E (*filled squares*) and for Ch4-2 (*open circles*) compared to Ch4-2 R591E (*filled circles*), using tail current data from *panel a* traces after leak-subtraction and normalization to maximal amplitude determined from Boltzmann equation fit (*curves*, see “Methods” section). HCN2 and Ch4-2 bind endogenous cAMP, whereas the corresponding R591E channels are unliganded. Boltzmann fit parameters of the representative curves are as follows: HCN2, − 83.8 mV, *s* = 12.5 mV; HCN2 R591E, − 98.2 mV, *s* = 11.4 mV; Ch4-2, − 93.3 mV, *s* = 19.8 mV; Ch4-2 R591E, − 109.1 mV, *s* = 14.7 mV. See Online Resource 1 for mean values

We first tested whether interactions between the HCN2 TM region and the C-terminal region influence cAMP potentiation. We constructed a chimeric channel called Ch4-2 by replacing the TM region of mouse HCN2 with that of mouse HCN4—a substitution which we term an "HCN4 TM-replacement". Channels were expressed as homomers and studied with two-electrode voltage clamp in intact *X. laevis* oocytes. Endogenous cAMP binds HCN channels in these cells (Dascal 1987; Wang et al. 2001; Magee et al. 2015), so to study unliganded channels we introduced a previously characterized mutation (R591E) in the CNB fold, eliminating an arginine required for contacting cAMP's cyclic phosphate (Chen et al. 2001b; Magee et al. 2015). Details of kinetics are addressed in later sections; we note qualitatively here that the HCN4 TM-replacement slowed gating, but cAMP-dependent speeding of activation was still apparent (Fig. 1a*, starred traces*). The *V*_1/2_ values of Ch4-2 and Ch4-2 R591E were each approximately 10 mV more negative than *V*_1/2_ of the corresponding HCN2 channel so that the cAMP-dependent *V*_1/2_ shift of Ch4-2 channels matched that of HCN channels within the experimental uncertainty (Fig. 1a, b). Specifically, in intact oocytes it was previously found that *V*_1/2_ of HCN2 R591E was − 99.4 ± 3.1 mV (*n* = 10) and *V*_1/2_ of liganded HCN2 was − 83.4 ± 5.0 mV (*n* = 49), indicating a cAMP-dependent *V*_1/2_ shift of + 15.9 ± 1.2 mV (Magee et al. 2015). For the new Ch4-2 channels, *V*_1/2_ of Ch4-2 R591E was − 107.9 ± 5.0 mV (*n* = 16) and *V*_1/2_ of Ch4-2 was − 93.2 ± 3.2 mV (*n* = 8), indicating a cAMP-dependent *V*_1/2_ shift of + 14.7 ± 1.7 mV. While our testing was limited to the subsaturating endogenous level of cAMP (Wang et al. 2001; Magee et al. 2015), we found no evidence to suggest that the HCN4 TM-replacement disrupts the cAMP-dependent *V*_1/2_ shift.

**Fig. 2:**
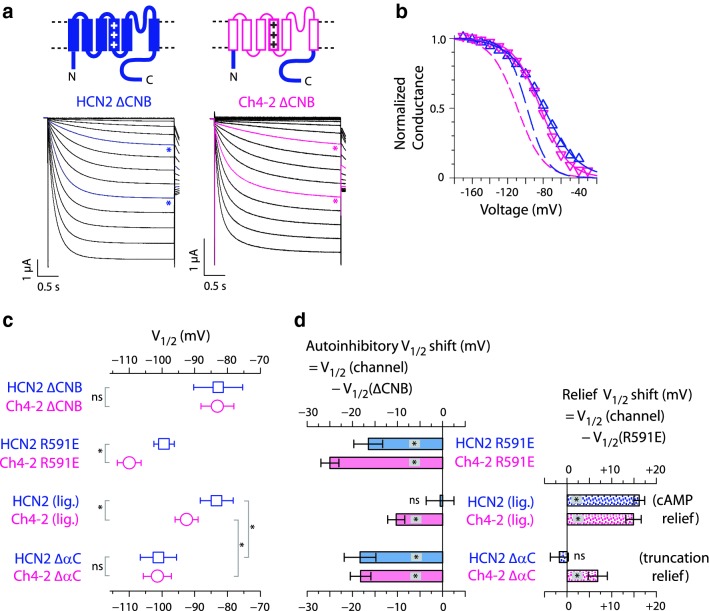
HCN4 TM-replacement in truncated HCN2 derivatives does not negatively shift *V*_1/2_. **a** Schematics: composition of ΔCNB channels truncated after the C-linker. Traces: representative inward currents of HCN2 ΔCNB (from Magee et al. 2015) and Ch4-2 ΔCNB, formatted as in Fig. 1a. **b** Conductance–voltage relationships for HCN2 ΔCNB (point-up triangles) and Ch4-2 ΔCNB (point-down triangles) using data from **a** and constructed as in Fig. 1b. For comparison with fully autoinhibited channels, the relations for HCN2 R591E (long-dash curve) and Ch4-2 R591E (short-dash curve) are shown repeated from Fig. 1b. Boltzmann fit parameters of the representative curves are as follows: HCN2 ΔCNB, − 79.4 mV, *s* = 19.2 mV; Ch4-2 ΔCNB, − 83.2 mV, *s* = 15.4 mV. **c** Mean *V*_1/2_ values for full-length and truncated channels with the HCN2 TM region (squares) or the HCN4 TM region (circles). *Error bars* show SD, with > 6 recordings for each channel (see Online Resource 1). Selected pairwise comparisons are marked as either statistically significant (*p < 0.05) or not significant (*ns*). **d** Comparisons with ΔCNB and R591E channels. The autoinhibitory *V*_1/2_ shift of each channel (*left panel*) was calculated from that channel's mean *V*_1/2_ after subtracting the mean *V*_1/2_ of the corresponding ΔCNB channel (i.e., having the same TM region sequence). The relief *V*_1/2_ shift (right panel) of each channel was calculated from that channel's mean *V*_*1*/2_ after subtracting the mean *V*_1/2_ of the corresponding R591E channel. *Error bars* show uncertainties from error propagation using SEM as uncertainty of individual mean *V*_1/2_ values. Each bar is marked as either significantly different than zero (*p < 0.05) or not significantly different than zero (*ns*)

The 10-mV negative *V*_1/2_ shift resulting from HCN4 TM-replacement in both HCN2 and HCN2 R591E might arise from augmentation of CNB fold-mediated autoinhibition, or from alteration of gating mechanisms that do not involve the CNB fold at all; these possibilities are not mutually exclusive. To distinguish these effects, for each channel, we determined the negative *V*_1/2_ shift due to autoinhibition (the autoinhibitory *V*_1/2_ shift, distinct from the cAMP-dependent *V*_1/2_ shift) through comparison to the corresponding "ΔCNB" channel, which fully lacks autoinhibition due to truncation after the C-linker (Wainger et al. 2001). The autoinhibitory *V*_1/2_ shift was previously determined as − 16.5 ± 3.2 mV for HCN2 R591E in intact oocytes (Magee et al. 2015). We found that *V*_1/2_ of Ch4-2 ΔCNB was − 82.9 ± 5.2 mV (*n* = 11) (Fig. 2a-c), which means that Ch4-2 R591E displayed an autoinhibitory *V*_1/2_ shift of − 25.0 ± 2.0 mV, larger by 10 mV than that of HCN2 R591E (Fig. 2c). Moreover, *V*_1/2_ of Ch4-2 ΔCNB is not significantly different from that of HCN2 ΔCNB (Fig. 2c) which suggests that the 10-mV negative shift of *V*_1/2_ after HCN4 TM-replacement in full-length channels is not caused by altering any regulatory mechanisms that operate in the absence of a CNB fold. This supports the notion that full-length HCN2 channels experience some sort of restriction in the magnitude of their autoinhibitory *V*_1/2_ shift, and this restriction is disrupted by HCN4 TM-replacement resulting in augmentation of the autoinhibitory *V*_1/2_ shift. Thus the *V*_1/2_ value for the cAMP-liganded Ch4-2 in intact oocytes can be explained by a sum of three contributions shown in Fig. 2d: conventional autoinhibition like HCN2, an augmented autoinhibition component absent from HCN2, and cAMP-dependent potentiation like HCN2. The conventional autoinhibition contribution would be sufficient for autoinhibitory *V*_1/2_ shift of 15 mV (Fig. 2d left panel, HCN2), but the augmented autoinhibition contribution supplements this by 10 mV, giving net autoinhibitory *V*_1/2_ shift of − 25 mV (Fig. 2d left panel, Ch4-2 R591E). The cAMP-dependent *V*_1/2_ shift of + 15 mV (Fig. 2d right panel, “cAMP relief”), derives from the conserved HCN2 C-terminal region and is not sensitive to HCN4 TM-replacement.

### Augmentation of Autoinhibitory *V*_1/2_ Shift can be Disrupted by Truncation After the Beta-roll of the CNB Fold

Previous studies identified the beta-roll of the CNB fold as a key structure for autoinhibition by characterizing a channel called HCN2 ΔαC which was truncated after the N-terminal moiety of the CNB fold (called the "beta-roll"); this lacked the terminal helix αC critical for cAMP potentiation, as well as the extreme C-terminal region (Wainger et al. 2001). HCN2 ΔαC channels are unliganded and therefore are autoinhibited (Wainger et al. 2001; Magee et al. 2015). But there is evidence that this truncation significantly alters the conformation of the C-linker and/or beta-roll, since HCN2 ΔαC channels do not fully support the Quick-Activation mechanism that controls activation kinetics in unliganded full-length HCN2 channels (Magee et al. 2015). So, we investigated whether or not the beta-roll was sufficient to support the augmented autoinhibition observed in Ch4-2. To test this, we performed the HCN4 TM-replacement in HCN2 ΔαC to make Ch4-2 ΔαC and determined its autoinhibitory *V*_1/2_ shift by comparison with Ch4-2 ΔCNB. The *V*_1/2_ value for Ch4-2 ΔαC was − 101.2 ± 4.5 mV (*n* = 7) (Fig. 2c, d), which means that Ch4-2 ΔαC channels displayed an autoinhibitory *V*_1/2_ shift of − 18.2 ± 2.3 mV. This is a substantially smaller autoinhibitory *V*_1/2_ shift that of Ch4-2 R591E and matches the conventional autoinhibitory *V*_1/2_ shift previously found (Magee et al. 2015) for HCN2 ΔαC (− 18.3 ± 3.5 mV; see Fig. 2d left panel). This suggests that augmented autoinhibition has been disrupted by the truncation after the beta-roll, producing approximately 10 mV of autoinhibition relief relative to Ch4-2 R591E (Fig. 2d right panel, "truncation relief"). In other words, the beta-roll of the CNB fold in the absence of helix αC or the extreme C-terminal region is not sufficient for augmentation of autoinhibitory *V*_1/2_ shift in presence of the HCN4 TM region, rather producing only the conventional autoinhibitory *V*_1/2_ shift with either the HCN2 or HCN4 TM region.

### HCN4 TM-Replacement Augments the Autoinhibition-Mediated Speeding of Deactivation

Although *V*_1/2_ shifts are commonly used to quantify modulation of voltage-gating, the relationship of *V*_1/2_ to HCN channel energetics is complex because *V*_1/2_ reflects stabilities of multiple open and closed states instead of a single closed–open transition. In particular, a situation may arise where the pore-opening step is voltage-independent and the maximum open probability at saturating hyperpolarization (*P*_max_) is significantly less than 100%; in this situation, a shift in the equilibrium of the pore-opening step could alter *P*_max_ without an observable shift in *V*_1/2_ (Craven and Zagotta 2004). Therefore, the different autoinhibitory *V*_1/2_ shift we observed in HCN2 and Ch4-2 might arise due to a lower *P*_max_ in HCN2 rather than a difference in autoinhibition strength. To find further evidence for augmented autoinhibition strength in Ch4-2, we examined gating kinetics. Here we discuss deactivation kinetics quantified by *t*_1/2_ (time required for 50% deactivation completion); activation kinetics are discussed in the next section.

Two proposed models (Wicks et al. 2009, 2011; Magee et al. 2015) in which the CNB fold could govern HCN channel deactivation kinetics are autoinhibition and open-state trapping. In an autoinhibition model using a Leffler-type formulation (Leffler 1953), the transition state for a rate-limiting step in deactivation should experience stabilization (relative to the starting ground state for that step), that is some fraction of the stabilization experienced by the destination ground state upon imposition of autoinhibition. Therefore, channels with an intact CNB fold would have a kinetically destabilized open state (faster deactivation) compared to ΔCNB channels. The opposite is true in an open-state trapping model so that channels with an intact CNB fold have a kinetically stabilized open state (slower deactivation) compared to ΔCNB channels. Open-state trapping has been observed for cAMP-liganded HCN2 as well as for an unliganded HCN4 derivative with a mutation in S4 (Wicks et al. 2009, 2011; Magee et al. 2015). Thus, for each channel, we compared its deactivation kinetics with those of the corresponding ΔCNB channel to distinguish autoinhibition from open-state trapping effects.

**Fig. 3:**
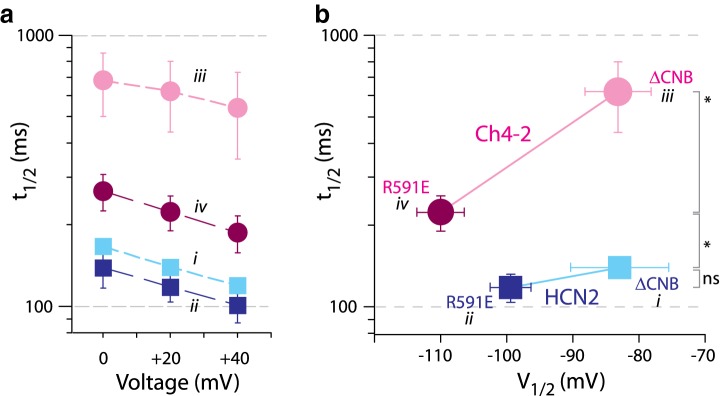
Effect of augmented autoinhibition on deactivation kinetics. **a** Mean *t*_1/2_ values with error bars showing SD, with ≥ 6 recordings for each channel. Datasets for HCN2 family channels are from Magee et al. (2015). Datasets are labelled with roman numerals for reference in **b**. Datasets are plotted for channels lacking cAMP potentiation: HCN2 ΔCNB, *light solid squares*, label *i*; HCN2 R591E, dark solid squares, label *ii*; Ch4-2 ΔCNB, light solid circles, label *iii*; Ch4-2 R591E, dark solid circles, label *iv*. **b** Correlation plot of deactivation *t*_1/2_ (+ 20 mV, from **a**) vs *V*_1/2_ (from Fig. 2c). Point symbols and roman numeral labels are as in **a**. The datapoints for ΔCNB and R591E channels are connected by a straight line representing autoinhibition. Pairwise comparisons were made using log t_1/2_ values and are marked as either statistically significant (*p < 0.05) or not significant (*ns*)

Ch4-2 R591E exhibited deactivation kinetics substantially slower than Ch4-2 ΔCNB, consistent with an autoinhibition model, and the magnitude of this speeding effect was strikingly much greater than that observed for HCN2 R591E. At each voltage tested ranging from 0 to 40 mV, Ch4-2 R591E deactivated faster than Ch4-2 ΔCNB by a factor of 2.6 or more (Fig. 3a, iii vs. iv), whereas HCN2 R591E deactivated faster than HCN2 ΔCNB by a factor of only 1.2-fold or less (Fig. 3a, i vs. ii). Since the *t*_1/2_ values exhibited depolarization dependence that was similar for HCN2 and Ch4-2 derivatives, we considered a simple hypothesis where the *t*_1/2_ vs. *V* relation of Ch4-2 R591E reflected a 25-mV negative shifting of the *t*_1/2_ vs. *V* relation of Ch4-2 ΔCNB, in parallel with the autoinhibitory *V*_1/2_ shift of 25 mV. But in fact, even a 40-mV negative shifting of the *t*_1/2_ vs. *V* relation of Ch4-2 ΔCNB would not be sufficient to explain the quantitative speeding of deactivation observed in Ch4-2 R591E. For instance, the *t*_1/2_ of Ch4-2 R591E at 0 mV was 2.0-fold lower (faster) than *t*_1/2_ of Ch4-2 ΔCNB at + 40 mV. The marked autoinhibitory speeding of deactivation in Ch4-2 R591E stands in strong contrast with HCN2 R591E, in which the identical C-terminal region sequence produces negligible autoinhibitory speeding of deactivation despite a prominent autoinhibitory *V*_1/2_ shift. Thus while the HCN2 C-terminal region in principle possesses a capability for substantial autoinhibitory speeding of deactivation, the intact HCN2 channel restricts this autoinhibition effect, just as it restricts the autoinhibitory *V*_1/2_ shift. This restriction is disrupted by HCN4 TM-replacement, implying a reliance on a functional interaction between the HCN2 TM region and C-terminal region. The underlying structural basis of this functional interaction might be a direct contact or might be indirect such as through a mediating structure like the N-terminal region (Porro et al. 2019).

### HCN4 TM-Replacement Slows Deactivation Kinetics Independently of Its Effect on Autoinhibition

Apart from its augmenting effect on autoinhibitory speeding of deactivation, HCN4 TM-replacement additionally produced a significant slowing influence on deactivation kinetics without involvement of the CNB fold. That is, HCN4 TM-replacement in HCN2 ΔCNB significantly increased *t*_1/2_ by a factor of 4.1 or more over all voltages tested (Fig. 3a, i vs. iii). Notably, HCN4 TM-replacement in HCN2 R591E increased *t*_1/2_ less markedly, by a factor of 1.9 or less. This can be explained because of the two opposing effects of HCN4 TM-replacement on deactivation kinetics: the fourfold slowing effect intrinsic to the HCN4 TM region is partially mitigated by the two to threefold speeding effect due to augmentation of autoinhibition. Stated another way, the different degrees of slowing from HCN4 TM-replacement in the ΔCNB and R591E sequence backgrounds provide evidence that the TM-replacement modified how the autoinhibitory C-terminal region governed deactivation kinetics.

Figure 3b provides a succinct visualization of autoinhibition magnitudes by plotting *t*_1/2_ (for + 20 mV deactivation) vs. *V*_1/2_ for R591E and ΔCNB channels. The Ch4-2 family of derivatives have generally higher *t*_1/2_ than HCN2 derivatives, reflecting an effect intrinsic to the HCN4 TM region. However, the magnitude of autoinhibition within each derivative family is represented by a correlation line drawn between the R591E and ΔCNB datapoints of the family. Compared to the HCN2 derivatives, the correlation line for the Ch4-2 derivatives is longer in both the horizonal and vertical dimensions, and moreover has a steeper slope. This illustrates how HCN4 TM-replacement in HCN2 R591E channels introduced an augmented contribution to autoinhibition which applies to both *V*_1/2_ and *t*_1/2_ compared to HCN2 channels, and also has a disproportionately large effect on *t*_1/2_ compared to *V*_1/2_.

**Fig. 4:**
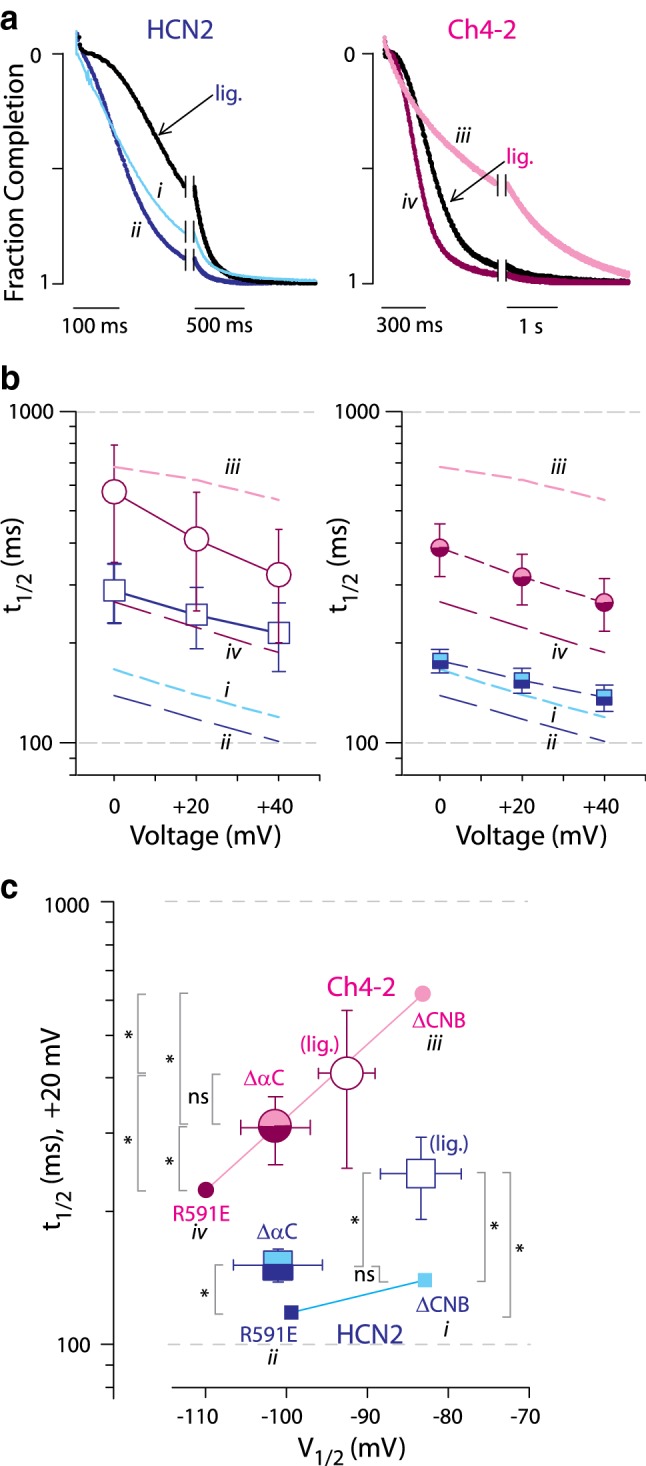
Effect of augmented autoinhibition on deactivation kinetics is partially relieved by cAMP binding or truncation within CNB fold. **a** Representative deactivation currents at + 20 mV, each normalized to total decay amplitude. Roman numeral labels are as in Fig. 3a. Recordings for HCN2 family channels are from Magee et al. (2015). Within each channel family, the maximally autoinhibited channel (full-length R591E, *ii* and *iv*) exhibits the smallest *t*_1/2_ values. For these example traces, the *t*_1/2_ are as follows: HCN2 ΔCNB (label *i*), *t*_1/2_ = 133 ms; HCN2 R591E (label *ii*), *t*_1/2_ = 119 ms; HCN2 lig., *t*_1/2_ = 255 ms; Ch4-2 ΔCNB (label *iii*), *t*_1/2_ = 632 ms; Ch4-2 R591E (label *iv*), *t*_1/2_ = 245 ms; Ch4-2 lig., *t*_1/2_ = 340 ms. **b** Mean *t*_1/2_ values with error bars showing SD, with ≥ 6 recordings for each channel; for some channels the bars are drawn wider for clarity. For reference, datasets are repeated from Fig. 3b for corresponding ΔCNB and R591E channels (dashed lines, labels *i* through *iv*). In *left panel,* datasets are plotted for channels with cAMP potentiation: HCN2, open squares; Ch4-2 open circles. In *right panel,* datasets are plotted for truncated channels: HCN2 ΔαC, half-shaded squares; Ch4-2 ΔαC, half-shaded circles. Datasets for HCN2 family channels are from Magee et al. (2015). **c** Correlation plot of deactivation *t*_1/2_ (+ 20 mV) vs. *V*_1/2_ is re-drawn from Fig. 3b with addition of cAMP-liganded and ΔαC channels. Point symbols and roman numeral labels are as in **b** for ΔCNB and R591E channels that define the correlation line (not a fit) for each derivative family. Pairwise comparisons were made using log *t*_1/2_ values and are marked as either statistically significant (*p < 0.05) or not significant (*ns*)

Sigmoidal shape with a delay phase is a characteristic feature of deactivation transients in HCN channels and is believed to reflect multiple sequential S4 movement steps in the voltage-sensing domain that precede the closed–open transition in the pore domain (Elinder et al. 2006; Kusch et al. 2010). Sigmoidicity was apparent in deactivation transients of HCN2 R591E and Ch4-2 R591E as well as the corresponding intact cAMP-liganded channels (Fig. 4a), but HCN2 ΔCNB and Ch4-2 ΔCNB showed reduced sigmoidicity. For instance, at early times, the fraction completion was greater for each ΔCNB channel than for the corresponding R591E channel (Fig. 4a). The reduced sigmoidicity in ΔCNB channels suggests that truncation after the C-linker loosened the requirement for multiple S4 movements before pore closure, albeit slowing overall progress through the deactivation pathway compared to the autoinhibited full-length R591E channels. This argues in support of the existence of functional interactions between the voltage-sensing region and the C-terminal region, where the C-terminal region structures that impose autoinhibition are also involved with imposing cooperativity between subunits of the tetramer.

### HCN4 TM-Replacement Leads to Kinetic Dominance of the Autoinhibition Mechanism During Deactivation

Partial relief of augmented autoinhibition, as evaluated by autoinhibitory *V*_1/2_ shift, can be achieved by either cAMP binding (liganded intact channels) or truncation following the beta-roll (ΔαC channels). If deactivation kinetics is also well described by an autoinhibition model, then t_1/2_ values of the cAMP-liganded Ch4-2 channel and of Ch4-2 ΔαC should be intermediate between those of Ch4-2 R591E and Ch4-2 ΔCNB. This prediction was borne out: over the voltages tested, liganded Ch4-2 deactivated at least 1.2-fold faster than Ch4-2 ΔCNB which indicates autoinhibition, but deactivated at least 1.7-fold slower than Ch4-2 R591E which demonstrates a slowing effect of cAMP potentiation on deactivation kinetics (Fig. 4a right, Fig. 4b left). Similarly, Ch4-2 ΔαC deactivated at least 1.8-fold faster than Ch4-2 ΔCNB but at least 1.4-fold slower than Ch4-2 R591E (Fig. 4b right). Figure 4c shows that even though the data for liganded Ch4-2 and Ch4-2 ΔαC were not used to determine the correlation line, their datapoints nonetheless fall on that line. The Ch4-2 derivatives as a group therefore conform to a regular progression well described by the correlation line from no autoinhibition (ΔCNB) to maximal autoinhibition (R591E) with varying degrees of autoinhibition relief from partial truncation (ΔαC) or cAMP binding (Ch4-2 lig.).

The straightforward progression of Ch4-2 derivatives in Fig. 4c contrasts with the group of HCN2 derivatives, where datapoints for liganded HCN2 and HCN2 ΔαC do not conform to the correlation line defined by comparing HCN2 R591E to HCN2 ΔCNB. Liganded HCN2 deactivates markedly slower than HCN2 ΔCNB due to open-state trapping (Magee et al. 2015) (Fig. 4a left, Fig. 4b left, Fig. 4c “HCN2 lig.”). HCN2 ΔαC also deactivates slower than HCN2 ΔCNB despite exhibiting conventional autoinhibitory *V*_1/2_ shift. This suggests that truncation after the beta-roll enables simultaneous operation of the open-state trapping mechanism and autoinhibition mechanism with opposing influences on deactivation kinetics, but with slightly greater influence from open-state trapping than from autoinhibition. The deactivation kinetics of HCN2 R591E may also be governed by simultaneous operation of open-state trapping and autoinhibition mechanisms with opposing influences that are coincidently balanced, giving rise to *t*_1/2_ values that resemble those of HCN2 ΔCNB. All together these results suggest that HCN2 and Ch4-2 differ in their complexity of mechanistic contributions to deactivation kinetics. HCN2 possesses two distinct mechanisms of open-state trapping and autoinhibition which can operate simultaneously, whereas the HCN4 TM-replacement disrupted the open-state trapping mechanism, leaving autoinhibition (with partial relief by cAMP binding) to be the dominant mechanism contributed by the C-terminal region to determine deactivation kinetics.

### HCN4 TM-Replacement Leads to Kinetic Dominance of the Autoinhibition Mechanism During Activation

HCN channels exhibit hysteresis such that the deactivation pathway is not the reverse of the activation pathway (Männikkö et al. 2005; Wicks et al. 2009; Kusch et al. 2010). As with deactivation, there are two proposed models in which the CNB fold governs HCN channel activation kinetics: autoinhibition and Quick-Activation (Wainger et al. 2001; Magee et al. 2015). Compared to ΔCNB channels, channels with an intact CNB fold have activation that is either slower (autoinhibition model) or faster (Quick-Activation model). We tested whether HCN4 TM-replacement leads to the kinetic dominance of the autoinhibition mechanism during activation as it did for deactivation. The sigmoidal activation transients were fitted to a sum of two exponentials following a delay (Wang et al. 2001; Magee et al. 2015) (see Online Resource 2); since the second exponential component had negligible amplitude in some conditions, a weighted-average time constant (*τ*_w_) was used for all comparisons. Notably, the activation kinetics of liganded HCN2 channels cannot be tested in intact oocytes. This is because the cAMP affinity of closed channels is too weak to enable binding of the low endogenous concentration of cAMP; rather, cAMP binding occurs over the course of the activation epoch as channels reach the open state with strong cAMP affinity (Wang et al. 2001; Magee et al. 2015). Therefore, we limited our quantitative comparisons of activation to the constitutively unliganded channels (R591E, ΔαC, and ΔCNB).

**Fig. 5:**
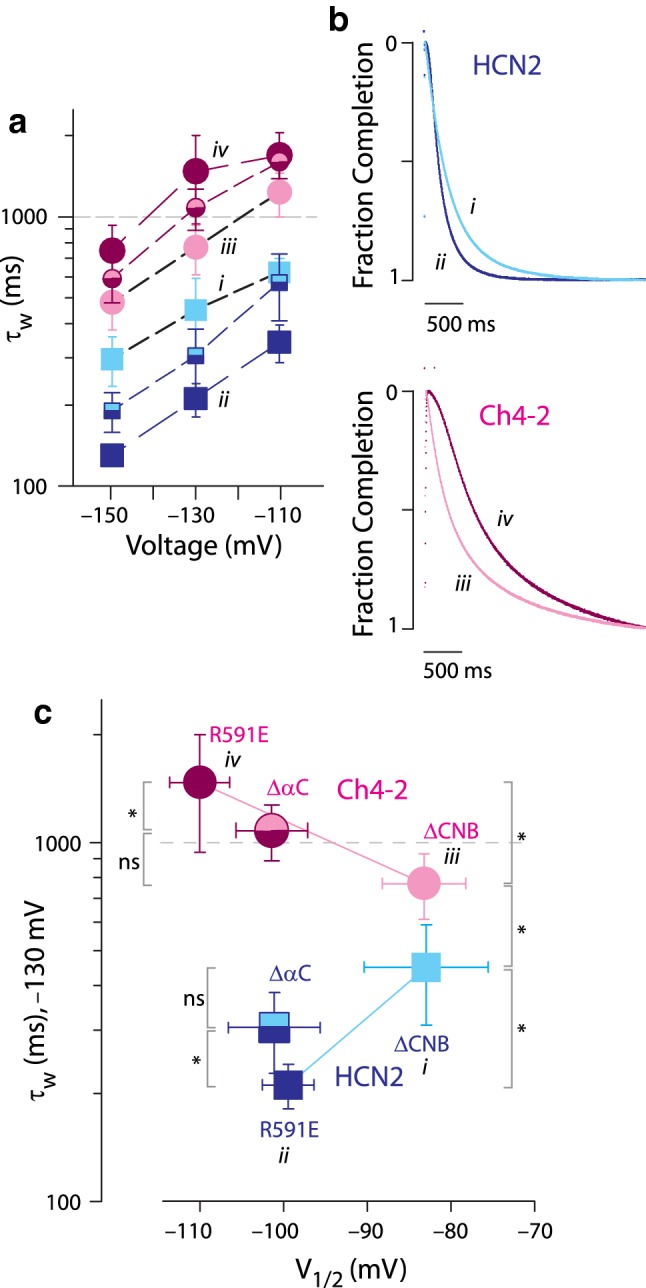
Effect of augmented autoinhibition on activation kinetics. **a** Mean *τ*_w_ values with error bars showing SD, with ≥ 6 recordings for each channel; for some channels the bars are drawn wider for clarity. Selected datasets are labelled with roman numerals for reference in **b** and **c**. Datasets for HCN2 family channels are from Magee et al. (2015). Datasets are plotted as follows: HCN2 ΔCNB, light solid squares, label *i*; HCN2 R591E, dark solid squares, label *ii*; HCN2 ΔαC, half-shaded squares; Ch4-2 ΔCNB, light solid circles, label *iii*; Ch4-2 R591E, dark solid circles, label *iv*; Ch4-2 ΔαC, half-shaded circles. **b** Representative activation currents at − 130 mV, each normalized to total decay amplitude in the 3-s activation epoch. Roman numeral labels refer to channels in **a**. HCN2 R591E (labelled *ii*) exhibits Quick-Activation, whereas Ch4-2 R591E (labelled *iv*) exhibits autoinhibition. Recordings for HCN2 family channels are from Magee et al. (2015). For these example traces, the lag (**d**) and *τ*_w_ values are as follows: HCN2 ΔCNB (label *i*), *d* = 120 ms and *τ*_w_ = 380 ms; HCN2 R591E (label *ii*), *d* = 150 ms and *τ*_w_ = 190 ms; Ch4-2 ΔCNB (label *iii*), *d* = 60 ms and *τ*_w_ = 680 ms; Ch4-2 R591E (label *iv*), *d* = 410 ms and *τ*_w_ = 1340 ms. Note the lack of sigmoidicity in ΔCNB channels indicated by smaller d values. **c** Correlation plot of activation *τ*_w_ (− 130 mV, from **a**) vs. *V*_1/2_ (from Fig. 2c). Point symbols and roman numeral labels are as in **a**. For each of the HCN2 and Ch4-2 groups of derivatives, the datapoints for ΔCNB and R591E channels are connected by a straight line (not a fit). Pairwise comparisons were made using log *τ*_w_ values and are marked as either statistically significant (*p < 0.05) or not significant (*ns*)

Over the voltages tested (− 110 to − 150 mV, Fig. 5a), HCN4 TM-replacement in HCN2 ΔCNB significantly increased *τ*_w_ by a factor from 1.6 to 2.0, indicating a slowing influence intrinsic to the HCN4 TM region (Fig. 5b, i vs. iii, Online Resource 1). The slowing effect of HCN4 TM-replacement was even more pronounced for longer HCN2 sequence backgrounds, with *τ*_w_ increasing at least 2.6-fold. As with deactivation, the different degrees of slowing from HCN4 TM-replacement in different sequence backgrounds indicates that the TM-replacement modified how the C-terminal region governed activation kinetics. Over the voltages tested, HCN2 R591E exhibited Quick-Activation, activating with *τ*_w_ at least 1.8-fold faster than HCN2 ΔCNB (Fig. 5a, b, i vs. ii). In contrast, Ch4-2 R591E exhibited autoinhibition of activation kinetics, activating with τ_w_ at least 1.4-fold slower than Ch4-2 ΔCNB (Fig. 5a, b, iii vs. iv). Additionally, Ch4-2 R591E showed longer delay phase (*d*, see Online Resource 2) than Ch4-2 ΔCNB due to markedly greater sigmoidicity in activation transients, suggesting loss of S4 cooperativity in ΔCNB channels as with deactivation. The findings suggest that the Quick-Activation mechanism of unliganded HCN2 was disrupted by the HCN4 TM-replacement, providing the first evidence that a key structural determinant of the Quick-Activation mechanism is in the HCN2 TM region.

The disruption of Quick-Activation can be visualized in the correlation plot of *τ*_w_ vs. *V*_1/2_ (Fig. 5c). The correlation lines drawn between ΔCNB and R591E datapoints have opposite slopes for Ch4-2 and HCN2, because Ch4-2 R591E exhibited autoinhibition of activation kinetics, whereas HCN2 R591E exhibited Quick-Activation. For each of Ch4-2 ΔαC and HCN2 ΔαC, the activation kinetics were intermediate between the corresponding ΔCNB and R591E channels. Notably, HCN2 ΔαC exhibited a weaker degree of Quick-Activation compared to HCN2 R591E, despite these two channels having the same autoinhibitory *V*_1/2_ shift (Magee et al. 2015). Consequently, in the correlation plot Fig. 4c, the HCN2 ΔαC datapoint falls well above the correlation line defined by ΔCNB and R591E channels. This indicates a complex combination of opposing gating characteristics in HCN2—an autoinhibitory *V*_1/2_ shift which disfavours activation, yet speeding of the rate-limiting step(s) for activation as indicated by *τ*_w_. In contrast, the Ch4-2 derivatives follow a straightforward progression in the correlation plot even though the Ch4-2 ΔαC datapoint was not used to derive the correlation line. Therefore, HCN4 TM-replacement had the effect of simplifying the complex gating of HCN2 so that autoinhibition was the dominant mechanism contributed by the C-terminal region to determine activation kinetics, just as for deactivation kinetics.

## Discussion

### Augmentation of Autoinhibition

Autoinhibition of HCN channels is mediated by the cytoplasmic CNB fold, but this study shows for the first time that the effects of this autoinhibition are not solely determined by the sequence of the C-terminal region but also can be strongly altered by substitution of the TM region. We analysed autoinhibitory *V*_1/2_ shift separately from cAMP-dependent *V*_1/2_ shift to determine the effect of TM-replacement on each one. This approach revealed that while HCN2 exemplified conventional autoinhibitory *V*_1/2_ shift, HCN4 TM-replacement introduced a substantial augmentation of this autoinhibitory *V*_1/2_ shift. This augmentation also relied on structures in the C-terminal region since it was abolished by truncation within the CNB fold after the beta-roll. We found further evidence for augmentation of autoinhibition in both activation kinetics (testing only unliganded channels) and deactivation kinetics (testing both unliganded and liganded channels). As a general rule, HCN4 TM-replacement slowed both activation and deactivation kinetics in either full-length or truncated HCN2 sequence backgrounds. But in full-length channels, HCN4 TM-replacement additionally augmented autoinhibition to significantly speed deactivation and slow activation (relative to the ΔCNB truncated channel). Augmentation of autoinhibition was so strong that the Quick-Activation and open-state trapping mechanisms of HCN2 were disrupted; autoinhibition hence became the dominant mechanism contributed by the C-terminal region to determine activation kinetics and deactivation kinetics.

As was found for *V*_1/2_, the effect of augmented autoinhibition on kinetics originated with introduction of the HCN4 TM region yet relied on structures in the C-terminal region. Notably, while augmented autoinhibition results from the combination of the HCN4 TM region and HCN2 C-terminal region, we are not suggesting that intact HCN2 has no functional interaction at all between these two regions. Rather, we propose that HCN2 uses a subtype-specific functional interaction between the TM and C-terminal regions to impose a restriction on autoinhibition strength. This interaction determines the magnitude of its autoinhibitory *V*_1/2_ shift; it also decides how much autoinhibition contributes to kinetics, relative to the restricting (opposing) contributions from open-state trapping for deactivation and from Quick-Activation for activation. Our study does not aim to distinguish whether autoinhibition affects S4 movement, gate movement, or the strength of S4-gate coupling, which are detailed features of voltage-gating governed by the TM region. Our concern here is the definition of structural requirements for CNB fold-dependent mechanisms—in particular, showing that some required structural components must reside in the TM region outside the CNB fold. The autoinhibitory action of the cytoplasmic C-terminal region in HCN channels is thus not an all-or-nothing effect, but rather has an adjustable magnitude and exerts separate effects on *V*_1/2_, activation kinetics, and deactivation kinetics, depending on the TM residues governing interaction with the C-terminal region.

Our use of autoinhibitory *V*_1/2_ shift as a measure of autoinhibition strength relies on an assumption of high *P*_max_ for Ch4-2 ΔCNB and HCN2 ΔCNB such that *V*_1/2_ would be sensitive to shifts in pore-opening equilibrium. HCN2 was previously found to have a low *P*_max_ of 70%, based on analysis of current amplitude and variance in excised membrane patches before and after cAMP application (Craven and Zagotta 2004; Johnson and Zagotta 2005). But in that cell-free configuration, loss of intracellular factors such as phosphatidylinositol 4,5-bisphosphate (PIP_2_) causes a decrease in HCN channel activity ("rundown") as evidenced by *V*_1/2_ values being generally more hyperpolarized than in the intact oocyte (Zolles et al. 2006; Pian et al. 2006, 2007). A low *P*_max_ after rundown may have obscured *V*_1/2_ shifts in a previous study (Stieber et al. 2003) that found HCN4 TM-replacement did not significantly change *V*_1/2_ in either full-length or truncated HCN2 sequence backgrounds; that study used human cells tested with the whole-cell patch configuration which exchanges cytoplasmic contents with pipette solution. In contrast, *P*_max_ is expected to be higher in our experiments using intact *X. laevis* oocytes tested in the two-electrode configuration which retains cytoplasmic contents. Unfortunately, the approaches for *P*_max_ determination in excised patches (Craven and Zagotta 2004; Johnson and Zagotta 2005) are not feasible in intact oocytes due to the much larger currents and the inability to perfuse the cytoplasmic membrane face, so high *P*_max_ must be taken as an assumption in our interpretation.

The above limitation to our interpretation of autoinhibitory *V*_1/2_ shift does not apply to our evaluation of kinetics that argues against an autoinhibition model, such as the findings that HCN2 ΔCNB does not have maximally fast activation or maximally slow deactivation, and that HCN4 TM-replacement augments the autoinhibition effect on deactivation kinetics. The slowing of *τ*_w_ we observed for full-length unliganded HCN2 R591E was similar in extent to that observed in a previous study (Stieber et al. 2003) of HCN4 TM-replacement in HCN2 (approximately threefold increase in activation time constant at − 140 mV for unliganded HCN2). Our study went beyond that previous study to compare our channels with ΔCNB channels, enabling us to test for the open-state trapping and Quick-Activation mechanisms.

### Correlation Between Kinetics and *V*_1/2_

The fundamental postulate of the autoinhibition model for CNB fold-mediated regulation is that the ΔCNB channel represents a minimal structural unit capable of voltage-gating with maximally favoured hyperpolarization-activation energetics. This postulate is supported by studies of mammalian HCN channel subtypes or mutated derivatives with varying magnitudes of cAMP-dependent *V*_1/2_ shift: it is consistently observed that the *V*_1/2_ value of a cAMP-liganded channel is never more positive than that of the corresponding ΔCNB channel (Wang et al. 2001; Wainger et al. 2001; Stieber et al. 2003; Wicks et al. 2011; Lolicato et al. 2011; Magee et al. 2015). The Ch4-2 family of channels exemplifies a Leffler-type application of this autoinhibition postulate to kinetics: the maximally positive *V*_1/2_ of the ΔCNB channel is associated with maximally fast activation kinetics, and conversely, maximally slow deactivation kinetics, and the autoinhibitory *V*_1/2_ shift of each longer derivative serves as a qualitative predictor of how much activation is slowed and deactivation is accelerated.

Correlation between *V*_1/2_ and kinetics is also well known for native HCN channels, where cAMP causes a positive *V*_1/2_ shift, speeding of activation kinetics, and slowing of deactivation kinetics (DiFrancesco and Tortora 1991; Ludwig et al. 1998, 1999; Santoro et al. 1998). However, it does not necessarily follow that the effects of cAMP on kinetics occur by virtue of a disruption of autoinhibition. The example of Quick-Activation and open-state trapping illustrates how the autoinhibition model fails to predict kinetics: the autoinhibition-free HCN2 ΔCNB channel with maximally positive *V*_1/2_ exhibits slower activation and faster deactivation than intact HCN2 channels. There is no contradiction in autoinhibition (governing *V*_1/2_) operating simultaneously with Quick-Activation and open-state trapping (governing kinetics). First, the Leffler assumption is not guaranteed to be valid, because the transition state for a conformational change may involve structural features that are not present in either the starting or destination ground states. Second, HCN channel gating uses a multi-step pathway (Craven and Zagotta 2004; Chen et al. 2007) so that the *V*_1/2_ reflects the entire set of ground states, whereas gating speed reflects transition state barrier height for only the rate-limiting step(s). Third, the hysteresis of HCN channels (Männikkö et al. 2005; Elinder et al. 2006; Wicks et al. 2011) means that the observed activation speed and deactivation speed may reflect wholly different transition states, such as S4 movement in closed channels for the activation pathway but S4 movement in open channels for the deactivation pathway. Our findings add to the growing body of evidence that channel activation and deactivation speeds should be examined separately from *V*_1/2_ trends to fully understand HCN channel behaviour.

### Possible Domain Interactions

Conventional autoinhibition in HCN channels relies strongly on interactions between C-linker regions in adjacent subunits of the tetramer (Ulens and Siegelbaum 2003; Craven and Zagotta 2004; Zhou et al. 2004). Such interactions are observable in the crystallographic and cryoEM structures of the unliganded isolated C-terminal region and full-length channel (Taraska et al. 2009; Lee and MacKinnon 2017, 2019). When the conformation of the C-linker is altered upon cAMP binding to the CNB fold or upon truncation of the CNB fold, the inhibitory interaction between C-linkers is believed to be disrupted (or converted to a different type of interaction) thus relieving autoinhibition. Our work shows that HCN4 TM-replacement has the effect of augmenting the conventional autoinhibitory *V*_1/2_ shift of HCN2, as well as magnifying the effect of autoinhibition on the rate-limiting steps for activation kinetics and deactivation kinetics. Since this augmentation of autoinhibition utilizes TM residues specific to HCN4, we suggest it has a different structural basis than conventional autoinhibition. We propose that HCN4 TM-replacement disrupted a key C-linker–TM region interaction used by HCN2 for restricting autoinhibition to prevent augmentation. In contrast, HCN4 TM-replacement left intact the C-linker–C-linker interaction responsible for conventional autoinhibition in HCN2. While interactions between the C-linker and TM region are also likely important for conventional autoinhibition, plausibly these are conserved during HCN4 TM-replacement since Ch4-2 ΔαC exhibited the same conventional autoinhibitory *V*_1/2_ shift as HCN2 ΔαC.

**Fig. 6:**
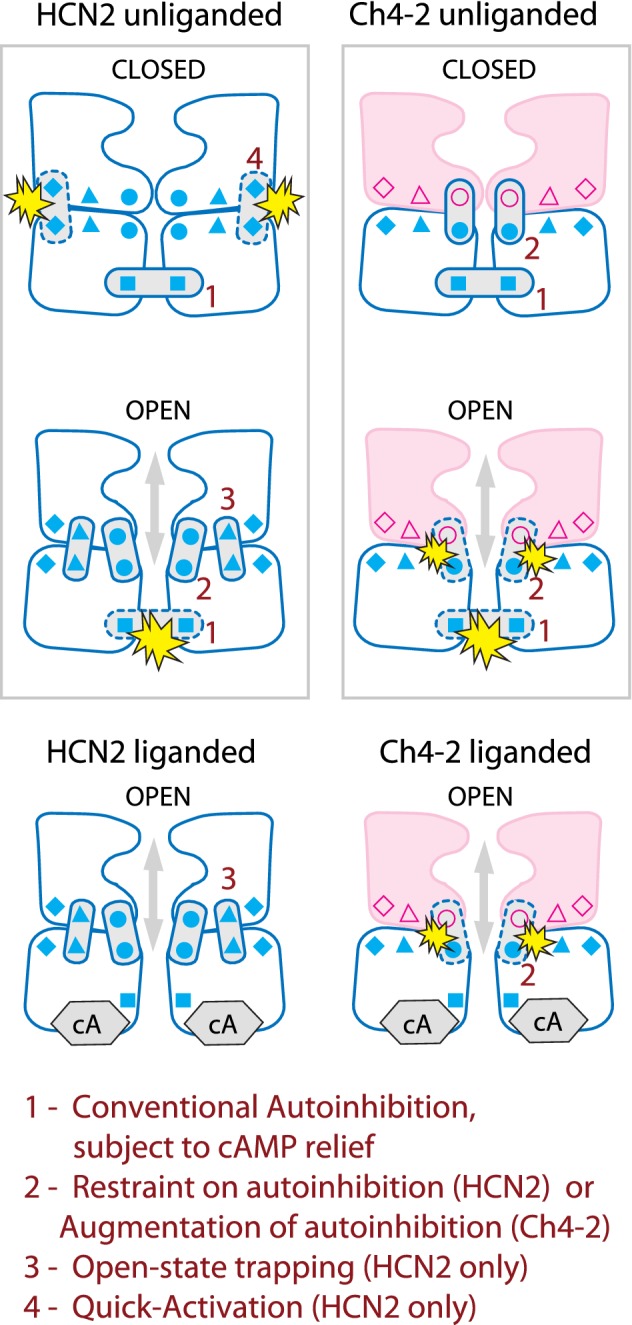
Summary of effects of HCN4 TM-replacement on CNB fold-mediated regulatory mechanisms of HCN2. Depictions of TM and C-terminal regions for two adjacent subunits in the tetramer are schematic and not intended to portray structural details. Interdomain interactions are represented by corresponding pairs of symbols joined by *bars*, with *solid-outline bars* for stabilizing interactions and *dashed-outline bars with starburst flare* for destabilizing interactions. The four types of functional interdomain interaction represented are conventional autoinhibition (*1*, squares), augmented autoinhibition (*2*, circles), open-state trapping (*3*, triangles), and Quick-Activation (*4*, diamonds). Open-state trapping and Quick-Activation affect only the rate-limiting step(s) for deactivation and activation pathways, respectively

Figure 6 summarizes schematically the multiple CNB fold-mediated mechanisms of HCN2 that are altered by HCN4 TM-replacement. For simplicity, the description here focuses on the C-linker of HCN2 but this is intended to encompass any indirect interactions mediated by the invariant HCN2 N-terminal "HCN domain" (Porro et al. 2019); the schematic also for clarity omits any C-linker–TM interactions that were conserved upon HCN4 TM-replacement. Conventional autoinhibition arises from a C-linker–C-linker interaction (Fig. 6 interaction 1, stabilizing closed state and destabilizing open state), but autoinhibition magnitude is restricted in HCN2 by a C-linker–TM interaction (Fig. 6 interaction 2, stabilizing open state). This restriction is disrupted by loss of the required TM residue in HCN4 TM-replacement, enabling the augmented component of autoinhibition (Fig. 6 interaction 2 in Ch4-2, stabilizing closed state and destabilizing open state). HCN4 TM-replacement also disrupts the open-state trapping and Quick-Activation of HCN2 (Fig. 6 interactions 3 and 4) leaving autoinhibition to be the predominant contribution governing the rate-limiting steps for both deactivation and activation pathways. Augmented autoinhibition in unliganded Ch4-2 is partially relieved when the C-linker rearranges as a result of either cAMP binding or partial CNB fold truncation. When cAMP binds to Ch4-2, the C-linker–C-linker interaction for the conventional component of autoinhibition is lost just as with HCN2 (Fig. 6 interaction 1 lost) but the C-linker–TM interaction for the augmentation component of autoinhibition in Ch4-2 would be retained (Fig. 6 interaction 2 retained). Truncation of the CNB fold after the beta-roll might represent a complementary situation, where the C-linker–C-linker interaction required for conventional autoinhibitory *V*_1/2_ shift is retained but the C-linker–TM interaction required for augmentation of autoinhibitory *V*_1/2_ shift is lost.

Within the C-linker, the A' helix immediately following S6 has been previously implicated in interactions with the TM region in determining open-state stability and *V*_1/2_ (Chen et al. 2001a; Decher et al. 2004; Kwan et al. 2012). The cryoEM structure of HCN1 showed multiple interactions of A’ helix residues with various parts of the TM region, including the conserved S4–S5 linker as well as more divergent parts like the S2–S3 linker which also extends into the cytosol. Some of these interactions were proposed to help stabilize the channel in the observed closed state, and to be altered upon binding cAMP (Lee and MacKinnon 2017). While *V*_1/2_ generally reflects relative stabilities of open vs. closed ground states, it is the gating kinetics (more than thermodynamics) that directly determines the contribution of HCN channels to rhythmic firing (DiFrancesco and Tortora 1991; Lüthi and McCormick 1998; Elinder et al. 2006). Our findings add to the growing body of evidence that channel activation and deactivation speeds should be examined separately from *V*_1/2_ trends to fully understand HCN channel behaviour. We consider it likely that some of the known physical interactions between the C-linker and the TM region could perform additional nuanced functions of controlling the magnitude of autoinhibition and the operation of Quick-Activation and open-state trapping. Specifically, potential interactions between divergent residues of the S2–S3 linker and the A’ helix, possibly in concert with the HCN2 domain, could control these important C-terminal mechanisms. Identification of these specific physical interactions could be explored in the future.

Since the A’ helix sequence is conserved in all mammalian HCN subtypes, the augmented autoinhibition observed in this biophysical study of homomeric channels could conceivably also operate in the heteromeric channels found in vivo that combine HCN4 with HCN2 or HCN1 (Much et al. 2003; Ye and Nerbonne 2009). This could have implications for truncations of the CNB fold in HCN4 found in patients with idiopathic sinus node dysfunction (Schulze-Bahr et al. 2003; Schweizer et al. 2010): the relief of autoinhibition caused by such truncations may be larger than predicted from in vitro studies on homomers of truncated HCN2 (Wainger et al. 2001; Magee et al. 2015).

## Materials and Methods

### Construct Composition

All chimera constructs (Ch4-2 series) include residues M1-G130 from mouse HCN2 (Santoro et al. 1998) fused to the transmembrane region M214-D521 of mouse HCN4 (Santoro and Tibbs 1999). This invariant sequence was then fused to various portions of the C-terminal region derived from mouse HCN2 (Magee et al. 2015): for Ch4-2, S444-L863; for Ch4-2 ΔαC, S444-E617; for Ch4-2 ΔCNB, S444-F525. The Ch4-2 R591E C-terminal region is identical to the Ch4-2 channel with the exception of the R591E mutation. All constructs were subcloned into the pGEM-HE vector for high expression in *Xenopus laevis* oocytes as previously reported for the corresponding HCN2 channel derivatives (Magee et al. 2015).

### Electrophysiology

As previously described (Magee et al. 2015), all channels were expressed as homomers from in vitro transcribed RNA injected into *X. laevis* oocytes, obtained using established procedures through ovariectomies following guidelines from the Canadian Council on Animal Care. Two-electrode voltage clamp recordings for each construct or condition were obtained from oocytes of at least three frogs. Each frog contributed no more than two data points per ovariectomy to overall average calculations for each Ch4-2 construct. As previously described (Magee et al. 2015), the bath solution (ND-96) consisted of (in mM) 96 NaCl, 3 KCl, 5 HEPES (pH 7.4), 1 MgCl_2_, and 0.75 CaCl_2_.

Oocytes were excluded if they exhibited inward leak (time-independent) current larger than 100 nA at the holding voltage of − 40 mV; leak stability was verified with a “staircase” of steps to + 40, 0 and − 40 mV applied after each test sweep. The activation protocol stepped from the holding voltage to voltages between + 20 and − 170 mV (Δ10 mV) for a 3-s activation epoch, followed by a tail epoch at − 120 mV and a 4-s deactivation epoch at + 20 mV. As described previously (Magee et al. 2015), late in the activation epoch at extreme hyperpolarizing voltages, some oocytes exhibited currents not typical of HCN channels; this atypical behaviour included a decrease in inward current or a second inflection point leading to concave-negative curvature. Individual sweeps at − 150 mV or more negative were excluded from analysis if they had atypical behaviour. Oocytes were excluded from analysis entirely if atypical behaviour was exhibited at − 140 mV or less negative.

The deactivation protocol stepped from the holding voltage to − 130 mV for a 4-s activation epoch, followed by a step to voltages between 0 and + 40 mV (Δ20 mV) for a 4-s deactivation epoch. The deactivation epoch was followed by a tail epoch at − 120 mV and a second deactivation epoch at 0 mV for 5 s. This second deactivation epoch was performed to ensure all channels are in the closed state before returning to the holding voltage. Channel behaviour was typically stable over at least 10 min, as verified with a control protocol which was identical to the deactivation protocol except it had a single sweep with 0 mV in the deactivation epoch. Oocytes were excluded from analysis if absolute tail currents differed by more than 100 nA in control protocols before and after a deactivation protocol.

### Data Analysis

Isochronal *V*_1/2_ (3-s activation epoch) was determined as described previously (Magee et al. 2015) using the four-parameter sigmoid Boltzmann equation to fit tail currents (*I*) vs. activation voltage (*V*):

$$ I = y_{0} + a/(1 + e^{{ - \left( {V - V_{1/2} } \right)/s}} ) $$

where *a* is maximum time-dependent HCN current amplitude (positive), *s* is reciprocal slope, *V*_1/2_ is midpoint activation voltage, and *y*_0_ is total maximum current (negative).

Deactivation transients reached endpoint consistently in the 4-s deactivation epoch, and the midpoint time of deactivation (*t*_1/2_) was determined as previously described (Magee et al. 2015).

Activation time constants following a delay (*d*) were determined as described previously (Wicks et al. 2009; Magee et al. 2015) using an iterative fit procedure with a double exponential fit equation:

$$ I\left( t \right)\; = \; A_{{{\text{early}}}} \; e^{{ - t/\tau_{{{\text{early}}}} }} \; + \;A_{{{\text{late}}}} \;e^{{ - t/\tau_{{{\text{late}}}} }} \; + \;C $$

The weighted-average time constant (τ_w_) was then calculated using the following equation:

$$ \tau_{{\text{w}}} \; = \;\left( {\tau_{{{\text{early}}}} *f_{{{\text{early}}}} } \right)\; + \;\left( {\tau_{{{\text{late}}}} *\left[ {1 - f_{{{\text{early}}}} } \right]} \right) $$

where *f*_early_ = *A*_early_/(*A*_early_ + *A*_late_).

Mean values are reported ± SD where *n* is the number of oocytes recorded. Comparisons of *V*_1/2_ values among channel derivatives were assessed by ANOVA with a significance threshold of *p* = 0.05, followed by post hoc Tukey’s test to identify significant pairwise differences. Numerical differences between mean *V*_1/2_ values are reported ± [(SEM1)^2^ + (SEM2)^2^]^1/2^, where SEM1 and SEM2 are the standard error of the mean *V*_*1*/2_ values. For comparing kinetic parameters, log *t*_1/2_ and log *τ*_w_ values were used for the ANOVA and post hoc Tukey’s tests.

## Electronic supplementary material

Below is the link to the electronic supplementary material.

Supplementary file1 (PDF 54 kb)
