# Supplementary material for: Cytoplasmic Autoinhibition in HCN Channels is Regulated by the Transmembrane Region
Source: J Membr Biol. 2020 Mar 7;253(2):153–66. doi: 10.1007/s00232-020-00111-8 (PMC7150657; doi:10.1007/s00232-020-00111-8)
Supplement: Supplementary file 1 — Supplementary file1 (PDF 54 kb) [file 232_2020_111_MOESM1_ESM.pdf]

	Conductance-Voltage relation		Deactivation kinetics (+20 mV)	Activation kinetics (-130 mV)		N
**Construct**	**$V_{1/2}$ (mV**	**s (mV)**	**$t_{1/2}$ (ms)**	**d (ms)**	**τ_w (ms)**	
**HCN2**	−83.4 ± 5.0	14.4 ± 2.6	243 ± 51	103 ± 28	322 ± 90	49
**Ch4-2**	−93.2 ± 3.2	18.9 ± 1.4	410 ± 160	186 ± 35	980 ± 150	8
**HCN2 R591E**	−99.4 ± 3.1	10.7 ± 1.8	118 ± 14	153 ± 25	211 ± 30	10
**Ch4-2 R591E**	−107.9 ± 5.0	13.5 ± 0.6	223 ± 33	450 ± 100	1470 ± 530	16
**HCN2 ΔCNB**	−82.9 ± 7.4	19.6 ± 1.4	139.6 ± 5.5	142 ± 93	450 ± 140	6
**Ch4-2 ΔCNB**	−82.9 ± 5.2	19.0 ± 3.7	620 ± 180	48 ± 19	770 ± 160	11
**HCN2 ΔαC**	−101.1 ± 5.5	8.5 ± 3.1	151 ± 13	221 ± 32	305 ± 78	10
**Ch4-2 ΔαC**	−101.2 ± 4.5	14.9 ± 1.5	309 ± 54	314 ± 72	1080 ± 190	7

Online Resource 1: Gating parameters of HCN2 and Ch4-2 derivatives. Values are means ± SD. Number of recordings is indicated by N, except that for HCN2, deactivation kinetics were not determined for 10 recordings so that N = 39. Recordings of HCN2 family channels were previously reported in Magee et al. (2015)

Reference:

Magee KEA, Madden Z, Young EC (2015) HCN Channel C-Terminal Region Speeds Activation Rates Independently of Autoinhibition. J Membr Biol 248:1043–1060. https://doi.org/10.1007/s00232-015-9816-7

Construct	d (ms)	τ_{early} (ms)	τ_{late} (ms)	f_{early}	τ_W (ms)	n
HCN2	103 ± 28	144 ± 31	960 ± 270	0.79 ± 0.06	322 ± 90	49
Ch4-2	186 ± 35	254 ± 31	1480 ± 210	0.41 ± 0.03	980 ± 150	8
HCN2 R591E	153 ± 25	137 ± 30.	360 ± 140	0.62 ± 0.16	211 ± 30	10
Ch4-2 R591E	450 ± 100	430 ± 100	2170 ± 800	0.40 ± 0.05	1470 ± 530	16
HCN2 ΔCNB	142 ± 93	323 ± 70.	1300 ± 1100	0.83 ± 0.10	450 ± 140	6
Ch4-2 ΔCNB	48 ± 19	228 ± 64	1240 ± 240	0.47 ± 0.04	770 ± 160	11
HCN2 ΔαC	221 ± 32	231 ± 58	630 ± 290	0.73 ± 0.29	305 ± 78	10
Ch4-2 ΔαC	314 ± 72	315 ± 61	1560 ± 250	0.40 ± 0.03	1080 ± 190	7

Online Resource 2: Detailed exponential fit parameters for activation transients at −130 mV**.** Values are means ± SD. Number of recordings is indicated by N. One recording of HCN2, one recording of HCN2 ΔCNB, and two recordings of HCN2 ΔαC lacked a detectable second component so no τ_{early} value was assigned. Recordings of HCN2 family channels were previously reported in Magee et al. (2015)

Reference:

Magee KEA, Madden Z, Young EC (2015) HCN Channel C-Terminal Region Speeds Activation Rates Independently of Autoinhibition. J Membr Biol 248:1043–1060. https://doi.org/10.1007/s00232-015-9816-7
